# Patients’ Perspectives About the Treatment They Receive for Cardiovascular Diseases and Mental Disorders: Web-Based Survey Study

**DOI:** 10.2196/32725

**Published:** 2022-03-16

**Authors:** Philippe Courtet, Catherine Pecout, Anne-Félice Lainé-Pellet, Michael Chekroun, Charlotte Avril, Jean-Jacques Mourad

**Affiliations:** 1 Service d’Urgence et Post-Urgence Psychiatrique Centre Hospitalier Universitaire de Montpellier Montpellier France; 2 Viatris Pfizer Inc France Paris France; 3 Carenity ELSE CARE Paris France; 4 Département de Médecine Interne et Centre d'Excellence, Société Européenne d’Hypertension Groupe Hospitalier Paris Saint Joseph Paris France

**Keywords:** medication adherence, iatrogenic risk, online patient community, patient experience, chronic disease, cardiovascular disease, mental disorder, noncommunicable disease, adherence, risk, community, experience, cardiovascular, mental health, perspective, treatment, mobile phone

## Abstract

**Background:**

Noncommunicable disease (NCD)–related deaths account for 71% of deaths worldwide. The World Health Organization recently developed a global action plan to address the impact of NCDs, with the goal of reducing the number of premature NCD-related deaths to 25% by the year 2025. Appropriate therapeutic adherence is critical for effective disease management; however, approximately 30%-50% of patients with an NCD do not comply with disease management activities as prescribed. Web-based patient communities can represent platforms from which specific information on patients’ perception of treatment adherence can be gathered outside of a clinical trial setting.

**Objective:**

This study aims to better understand patients’ perspectives regarding therapeutic adherence and iatrogenic risk in 2 major groups of NCDs for which poor disease management can have fatal consequences: cardiovascular diseases and mental disorders. Therapeutic adherence, motivational factors, patients’ awareness and perception of iatrogenesis, and treatment tools used by patients were assessed.

**Methods:**

A web-based survey was performed among patients with cardiovascular diseases or mental disorders or both conditions who were registered on the French Carenity platform, a web-based community in which patients with an NCD can share experiences and receive support and information. The study inclusion criteria were defined as follows: diagnosis of cardiovascular disease or mental disorder or both conditions (self-declared), age ≥18 years, residence in France, registration on the French Carenity platform, and ongoing pharmaceutical treatment for the condition. Patients who met the inclusion criteria were then invited to complete a self-administered web-based questionnaire that included questions addressing therapeutic adherence and iatrogenic risk.

**Results:**

A total of 820 patients were enrolled in the study, including patients with cardiovascular diseases (403/820, 49.2%), patients with mental disorders (292/820, 35.6%), and patients with both cardiovascular diseases and mental disorders (125/820, 15.2%). The mean age of the participants was 55.2 (SD 12.7) years. We found that 82.8% (679/820) of patients experienced adverse effects of medication. Patients tended to perceive themselves to be more adherent than they actually were; a significant number of patients disregarded their prescription and stopped or interrupted medication without consulting with a doctor. Patients with cardiovascular diseases were nearly twice as adherent as patients with a mental disorder (P≤.001). Adherence was significantly associated with gender (P≤.001), age (P≤.001), and treatment complexity (P≤.001). Finally, for each disease type, 3 patient profiles were identified, which provide interesting insight for improving therapeutic adherence and adjustment strategies specifically according to patient behavior.

**Conclusions:**

This study provides insight into the perspectives of patients receiving therapy for cardiovascular diseases or mental disorders or both conditions, which could help improve the management of NCDs and prevent premature death. Our study also shows that web-based patient platforms provide new opportunities to improve disease management by understanding patients’ experiences.

## Introduction

Deaths from noncommunicable diseases (NCDs), also known as chronic diseases, account for approximately 41 million deaths each year and 71% of deaths worldwide [1]. The World Health Organization recently developed a global action plan to address the impact of NCDs, with a goal of reducing premature NCD-related deaths to 25% by 2025 [2]. As premature deaths due to NCDs are avoidable, many policy recommendations have been proposed to prevent NCDs and improve disease management for patients with NCDs.

Cardiovascular disease–related deaths account for the majority of NCD-related deaths and 30% of global mortality [3]. Mental disorders also represent a major group of NCDs and account for approximately 14% of the global disease burden [4]. Cardiovascular diseases and mental disorders are major economic burdens on health care systems in terms of the direct (eg, medical consultations, hospitalizations, rehabilitation services, and medications) and indirect (eg, loss of productivity and short- or long-term disability) costs associated with mortality and morbidity [4-6].

Appropriate therapy management, including medication adherence, is critical for effective disease management and for improving patients’ overall quality of life [7,8]. However, approximately 30%-50% of patients with an NCD do not comply with disease prevention and management activities [9,10] such as following treatment as prescribed by a physician, staying up-to-date with medical appointments, engaging in regular physical activity, and making necessary dietary changes [7,11]. Medication adherence is defined as the extent to which patients take medications as prescribed in agreement with their health care provider [12]. Poor therapeutic adherence, which has significant effects on treatment outcome and disease prognosis [13], is driven by many factors such as limited disease awareness, poor understanding of the benefits and efficacy of prescribed regimens, and perceived or actual barriers (eg, adverse effects, financial constraints) [7,9,13,14]. Poor disease management can have fatal consequences, especially in patients with cardiovascular diseases and mental disorders [2,15,16]. Furthermore, patients with an NCD are at risk of iatrogenic disease, which is any pathologic condition caused by adverse medication reactions or complication induced by nondrug medical interventions, including diagnosis, intervention, error, and negligence. Although iatrogenic diseases can have major psychomotor and social consequences, most of these are avoidable with close disease management [17,18].

Few studies have been performed on therapeutic adherence from the patient point of view, which could provide valuable insight into patients’ perspectives and experiences for improving NCD management. As patients involved in adherence studies are often not representative of the general patient population for a given disease, bias may limit result extrapolation. However, web-based platforms on which patients can obtain information and share their medical experiences anonymously, without medical supervision, may more accurately represent patients’ perspectives and behaviors.

Web-based patient platforms such as registries, forums, social networks, and web-based communities offer patients the opportunity to participate in scientific studies and voluntarily share experiences regarding treatment benefits and burdens outside of the clinical setting [19]. Such information provides researchers with a better understanding of patients’ experiences, expectations, and unmet needs [19]. The insight gained from these web-based resources may also enhance clinical decision making, study protocol development, and patient recruitment [19]. The Carenity platform is an international web-based community for patients with chronic diseases and their caregivers. The platform provides an environment for patients to share their experiences, monitor their health, provide support, and contribute to medical research through web-based surveys [20]. Currently, over 400,000 patients (88%), primarily with chronic diseases, and their caregivers (12%) from 6 countries (France, Italy, Germany, Spain, the United Kingdom, and the United States) are registered on the platform.

Most studies on therapeutic adherence have been based on in-person interviews. However, under these conditions, patients may be reluctant to report poor medication adherence to avoid disappointing their physician. As social media networks represent useful and valuable resources for patients to obtain medical information and share their experiences with other patients, a web-based survey of patients with cardiovascular diseases or mental disorders or both conditions was performed to better understand patients’ perspectives of therapeutic adherence and iatrogenic risk. An anonymous and self-administered questionnaire was used to understand aspects of therapeutic adherence, including motivational factors, patients’ awareness and perception of iatrogenesis, and tools used by patients to facilitate adherence.

## Methods

### Study Design

A web-based survey of patients with cardiovascular diseases or mental disorders or both conditions was conducted. All patients were registered on the French Carenity platform, a web-based community in which both patients with chronic diseases and their caregivers can share their experiences, provide support, and share or receive information. A caregiver is defined as a person who provides care to someone with a chronic disease, disability, or other long-term health condition, typically outside a professional or formal framework. The community enhances patient-centered approaches by sharing patients’ experiences through web-based surveys, in which members may voluntarily participate. In February 2020, approximately 7600 members with at least one mental disorder and 10,000 members with at least one cardiovascular disease were registered on the platform.

### Participant Recruitment

Patients were recruited from February 14, 2020, to May 15, 2020, via invitation and follow-up no-reply emails. The study inclusion criteria were defined as follows: diagnosis of cardiovascular disease or mental disorder or both conditions (self-declared), age ≥18 years, residence in France, registration on the French Carenity platform, and ongoing pharmaceutical treatment for the condition. Patients who met the inclusion criteria and agreed to receive invitations were then contacted to complete a self-administered web-based questionnaire available on the Carenity website and promoted on Facebook. Patients who completed the questionnaire but did not meet the inclusion criteria were screened out, and patients who did not finish the questionnaire were not included in the analysis.

### Data Collection

The questionnaire comprised 39 questions regarding sociodemographic and medical information, including questions addressing therapeutic adherence and iatrogenesis risk. The questionnaire was developed and approved by a multidisciplinary board of experts, including a psychiatrist and a cardiovascular specialist. It was also approved by 2 Carenity members to ensure that the proposed questions were appropriate for the target audience. Data collected on the Carenity platform are hosted in France on a secured computer server in accordance with the Commission Nationale de l'Informatique et des Libertés, declaration number no 1484083, dated March 29, 2011.

#### Demographic and Clinical Characteristics

Information on demographic variables (eg, age, gender, education level, and professional status) and clinical characteristics (eg, disease type, age at diagnosis, comorbidities, and current treatments) was collected.

#### Treatment Complexity Score

The treatment complexity score was calculated by assigning 1 point for each constraining aspect of the pharmacological therapy, such as “varying number of medications each day,” “taking some medications with meals,” “taking some medications outside mealtimes,” “taking some medications at a set time each day,” “taking some medications on certain days and not others,” and “doses frequently change for some medications.” The treatment complexity score ranged from 0 (simplest treatment) to 7 (most complex treatment), and complexity was then classified into 3 categories based on the score: simple (score 0-1), intermediate (score 2), and complex (score 3-7).

#### Patient Lifestyle and Risk Level

Information was collected on the following 4 behavioral risk factors most associated with NCDs: tobacco use, excessive alcohol consumption, unhealthy diet, and physical inactivity [2]. Height and weight data were also recorded for each patient to calculate BMI. Nutritional status was classified based on patient BMI (weight [kg]/height squared [m^2^]) according to World Health Organization recommendations: underweight (BMI < 18.5), normal weight (BMI 18.5-24.9), overweight (BMI 25-29.9), and obese (BMI ≥ 30) [21].

Patients were then categorized into 3 risk level groups. The risk level of each patient was based on the following 5 risk factors: age ≥65 years, BMI ≥ 30, occasional or regular smoking, daily alcohol consumption or consumption of at least 6 drinks every week, and physical inactivity. The risk level was estimated by assigning 1 point per risk factor as follows: 0-1 risk factor, low risk; 2 risk factors, moderate risk; and 3-5 risk factors, high risk.

#### Iatrogenesis

To collect data on iatrogenic risk, questions focused on the adverse effects that patients experienced and the worries and fears that they had about their medication. Information was also collected on medications, including any interactions, adverse effects, benefits, and channels used to obtain information.

#### Medication Adherence

Perceived medication adherence was determined based on the response to the following question: “How well do you think you take your medications?” Patients were prompted to respond on a scale of 0 to 100 (0 corresponded to “I do not take my medications as prescribed” and 100 corresponded to “I take all my medications exactly as prescribed”).

Actual adherence was assessed based on the response to the questions considering all medications and not only to those for cardiovascular diseases or mental disorders. Participants were asked the following 4 questions: “Regarding your medications, do you ever (1) take medication late or early, (2) disregard the prescribed dose without medical advice, (3) intentionally stop/interrupt a treatment without medical advice, and (4) unintentionally stop/interrupt a treatment (because you forgot, etc.)?” Respondents were instructed to select the most appropriate response (never, very rarely, sometimes, often), with only 1 response per question. The adherence score was then calculated by assigning points according to the frequency of nonadherent behaviors. For the question about taking medication late or early, points were assigned as follows: 1 point for very rarely, 2 points for sometimes, and 4 points for often. For the questions about disregarding the prescribed dose and unintentionally stopping or interrupting treatment, points were assigned as follows: 2 points for very rarely, 4 points for sometimes, and 6 points for often. Finally, for the question on intentionally stopping or interrupting treatment, points were assigned as follows: 4 points for very rarely, 8 points for sometimes, and 12 points for often. Patients were then grouped into the following 4 compliance categories based on the total points: perfectly adherent (0 points), mostly adherent (1-2 points), partially adherent (3-7 points), and poorly adherent (≥8 points).

Information was also collected on adherence reporting, including frequency of doctor notifications and reasons patients did not consult with their doctors. Finally, data were also collected on solutions implemented by patients to improve disease management, such as tools used and types of assistance received, and additional patient needs.

### Statistical Analysis

Descriptive multivariable statistical analyses were performed. Categorical variables are expressed as absolute frequency and percentage. Continuous variable data are presented as the mean (SD) for normal distribution and as the median and interquartile range for non-normal distribution. A chi-square (*χ*^2^) test was applied to determine statistically significant differences for categorical variables, and a one-way analysis of variance or *t* test was applied to determine statistically significant differences for continuous variables.

A multiple correspondence analysis was performed for each of the 2 disease types to identify profiles of patients with similar therapeutic behaviors. To refine these profiles, 3 unsupervised classification models (ascending hierarchical classification, Kmeans, and partitioning around medoids algorithm) were compared using indicators of similarity and dissimilarity. The method with the best results was the 3-class ascending hierarchical classification model based on 12 variables covering the following 3 categories: demographic variables (gender and age), medical characteristics (eg, number of medications, adherence, adverse effects, treatment adjustment, and complementary therapies), and survey responses (fear of disappointing the physician, fear of risks associated with the therapy, discussions with the pharmacist, awareness of the risks of adverse effects, and risk of experiencing adverse effects). Data processing and analysis were performed using R (version 3.6.1; R Core Team).

Patients with cardiovascular diseases only were classified into 3 different profiles: at-risk, reporter, and tolerant. Patients with mental disorders only were also grouped into 3 different profiles: fearful, at-risk, and confident.

### Ethical Considerations

This survey was conducted in accordance with the Declaration of Helsinki and the principles of Good Clinical Practice. Prior to data collection, all patients provided consent on the Carenity website for the analysis of their anonymous health data for the study and subsequent publication of the findings (patients were informed that their health data would be collected and analyzed uniquely upon explicit consent, which was formalized by clicking the “Start” button at the bottom of the information page of the web-based survey). Participant privacy and confidentiality were guaranteed according to European laws and regulations (General Data Protection Regulation). As informed consent was provided by all patients prior to completing the survey, ethical review and approval were waived for this study.

## Results

### Respondent Profiles

**Figure 1:**
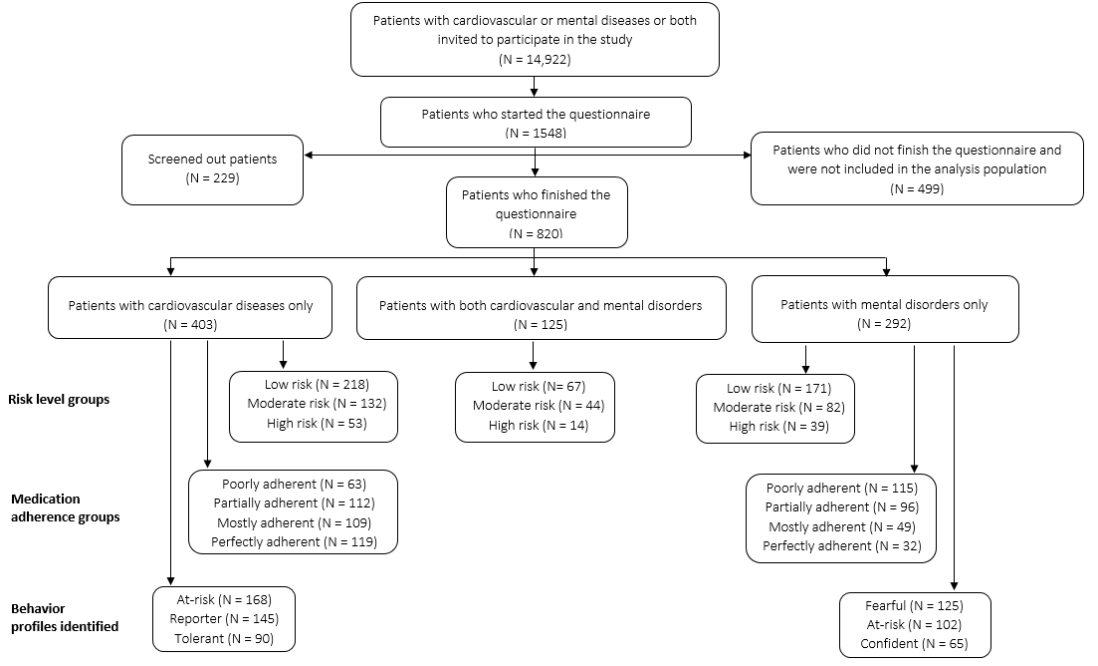
Study population flowchart from the screening of patients to the grouping according to risk level, medication adherence, and behavior profiles.

During the recruitment period, 820 patients with mental disorders or cardiovascular diseases or both conditions were enrolled in the study. Depending on the disease category, the 820 patients were recruited, selected, and grouped according to the risk level, therapeutic adherence, and behavior profiles as shown in Figure 1. Patients who answered being a “patient with cardiovascular disease or a cardiovascular risk factor” in the questionnaire were categorized in the “Patients with cardiovascular diseases only” group. The main diseases indicated by these patients in the questionnaire were high blood pressure (232/403, 57.6%), diabetes (120/403, 29.8%), and myocardial infarction (100/403, 24.8%).

Patients who answered being both a “patient with cardiovascular disease or a cardiovascular risk factor” and a “patient with a psychological disorder” in the questionnaire were categorized in the “Patients with both cardiovascular and mental disorders” group. The main diseases indicated by these patients in the questionnaire were high blood pressure (302/528, 57.2%), diabetes (170/528, 32.2%), and myocardial infarction (112/528, 21.2%). Patients who answered being a “patient with a psychological disorder” only in the questionnaire were categorized in the “Patients with mental disorders only” group. The main diseases indicated by these patients in the questionnaire were bipolar disorder (145/292, 49.7%), depression (135/292, 46.2%), and anxiety (125/292, 42.8%). In the risk level groups, the risk level of each patient was based on 5 determined risk factors and estimated by assigning 1 point per risk factor. Actual adherence was assessed based on the response to a set of 4 questions. The adherence score was then calculated by assigning points according to patients’ answers. Patients were then grouped into 4 compliance categories based on the total points. Profiles were identified by using a 3-class ascending hierarchical classification model based on 12 variables.

The study group consisted of 542/820 (66.1%) women and 278/820 (33.9%) men, with a sex ratio (male/female) of 0.5. Patients ranged in age from 18 to 93 years (mean 55.2 years, SD 12.7 years). The demographic characteristics of the patients are summarized in Table 1.

Regarding disease category, 49.2% (403/820) of patients had cardiovascular disease; the most common conditions reported were high blood pressure (302/528, 57.2%), diabetes (170/528, 32.2%), myocardial infarction (112/528, 21.2%), hypercholesterolemia (100/528, 18.9%), and arrhythmia (92/528, 17.4%). In total, 35.6% (292/820) of patients had a mental disorder; the most common conditions reported were depression (199/417, 47.7%), anxiety (193/417, 46.3%), and bipolar disorder (184/417, 44.1%). A total of 15.2% (125/820) of patients had both conditions. The medical characteristics of the patients are summarized in Table 2.

**Table 1:** Demographic characteristics of the patients with mental disorders or cardiovascular diseases or both conditions who were recruited for the web-based survey on the French Carenity platform.

Demographic characteristics			Total (N=820)	Patients with cardiovascular diseases only (n=403)				Patients with mental disorders only (n=292)				Patients with both conditions (n=125)				*P* value	
**Gender, n (%)**																	≤.001
	Female		542 (66.1)				217 (53.8)				228 (78.1)			97 (77.6)			
	Male		278 (33.9)				186 (46.2)				64 (21.9)			28 (22.4)			
**Age group (years), n (%)**																	≤.001
	≥65		189 (23)		149 (37)				19 (6.5)				21 (16.8)				
	<65		631 (77)			254 (63)				273 (93.5)				104 (83.2)			
Mean age (SD) (years)			55.2 (12.7)	60.6 (11)				47.6 (12.1)				55.9 (10.2)				N/A^a^	
**Level of education completed, n (%)**																	.30
		None	46 (5.6)				27 (6.7)				16 (5.5)			3 (2.4)			
		Elementary to middle school	71 (8.7)				38 (9.4)				21 (7.2)			12 (9.6)			
		High school	391 (47.7)				196 (48.7)				133 (45.5)			62 (49.6)			
		University	312 (38)				142 (35.2)				122 (41.8)			48 (38.4)			
**Professional status, n (%)**																	≤.001
		Currently working	309 (37.7)				142 (35.2)				135 (46.2)			32 (25.6)			
		Retired	247 (30.1)				179(44.4)				26 (8.9)			42 (33.6)			
		Other or not active	264 (32.2)				82 (20.4)				131 (44.9)			51 (40.8)			

^a^N/A: not applicable.

**Table 2:** Medical profile of the patients with mental disorders or cardiovascular diseases or both conditions who were recruited for the web-based survey on the French Carenity platform.

Medical characteristics				Total (N=820)		Patients with cardiovascular diseases only (n=403)	Patients with mental disorders only (n=292)			Patients with both conditions (n=125)		*P* value^a^	
**Time since diagnosis (years), n (%)**													.20
		0-5	193 (23.5)		113 (28)			67 (23)	13 (10.4)				
		5-20	330 (40.2)		166 (41.2)			118 (40.4)	46 (36.8)				
		>20	162 (19.8)		62 (15.4)			52 (17.8)	48 (38.4)				
		Do not remember	135 (16.5)		62 (15.4)			55 (18.8)	18 (14.4)				
Mean (SD) (years)				14.2 (11.7)		12.5 (11)	13.5 (10.6)			21.3 (13.5)		N/A^b^	
**Number of medications per day, n (%)**													≤.001
		1-2	263 (32.1)		113 (28)			130 (44.5)	20 (16)				
		3-5	312 (38)		158 (39.2)			112 (38.4)	42 (33.6)				
		≥6	245 (29.9)		132 (32.8)			50 (17.1)	63 (50.4)				
Mean (SD) number of medications per day				4.7 (3.71)		5.1 (4.17)	3.6 (2.7)			5.9 (3.6)		N/A	
**Number of pills per day, n (%)**													.08
		1	79 (9.6)		48 (11.9)			29 (9.9)	2 (1.6)				
		2-5	359 (43.8)		181 (44.9)			138 (47.3)	40 (32.)				
		≥6	380 (46.4)		173 (42.9)			125 (42.8)	82 (65.6)				
		Do not know	2 (0.2)		1 (0.3)			0 (0)	1 (0.8)				
Mean (SD) number of pills per day				6.4 (5.1)		6.1 (5.1)	5.9 (4.6)			8.8 (5.8)		N/A	
**Complexity of treatment regimen, n (%)**													.003
	Simple			269 (32.8)		148 (36.7)	97 (33.2)			24 (19.2)			
	Intermediate			204 (24.9)		104 (25.8)	66 (22.6)			34 (27.2)			
	Complex			347 (42.3)		151 (37.5)	129 (44.2)			67 (53.6)			
**Risk level (lifestyle), n (%)**													.40
		Low risk		456 (55.6)		218 (54.1)	171 (58.5)			67 (53.6)			
		Moderate risk		258 (31.5)		132 (32.7)	82 (28.1)			44 (35.2)			
		High risk		106 (12.9)		53 (13.2)	39 (13.4)			14 (11.2)			

^a^Cardiovascular diseases vs mental disorders.

^b^N/A: not applicable*.*

The majority of patients were polymedicated; only 14.1% (116/820) of patients were taking only 1 medication. The average number of medications per day was 4.7 (SD 3.7), with a mean of 6.4 (SD 5.1) pills per day per patient (Table 2). Thus, treatments were very burdensome for some patients who took ≥10 pills (168/820, 20.5%).

Moreover, patients >65 years of age were more often polymedicated and took >3 medications per day (116/189, 61.4%) with an average of 5.1 (SD 3.3) medications per day.

### Lifestyle and Risk Level

At the time of the survey, a quarter of patients (210/820, 25.6%) were smokers. Most patients (605/820, 73.8%) consumed alcohol, albeit at frequencies that varied between “a few times per year” (278/820, 33.9%) to “at least 3 drinks per day” (20/820, 2.4%).

The mean BMI was 29 (SD 7) kg/m^2^, and 69.1% (567/820) of participants were overweight or obese (BMI ≥ 25). Most patients (582/820, 71%) indicated that they did not adhere to any type of diet.

### Iatrogenesis

Regarding iatrogenic risk, 84.7% (695/820) of patients indicated worrying about risks associated with their medication; the main reasons were fear of dependence on treatment (372/695, 53.5%), lack of medication efficacy (373/695, 53.7%), the effect the medication will have on another condition (304/695, 43.7%), and intolerance or allergy to the medication (291/695, 41.9%). Less adherent patients are more likely to worry about dependency on treatment than perfectly adherent patients (89/199, 44.7% vs 36/128, 28.1%; *P*=.004). Nearly half of patients (432/820, 52.7%) worried about treatment risks and constraints when they started a new treatment; 73.1% (316/432) of these patients anticipated adverse effects.

A large majority of patients (722/820, 88%) worried about at least one treatment risk or constraint. These patients had discussed these issues with their general practitioner (307/722, 42.5%), a health specialist (305/722, 42.2%), or a relative (141/722, 19.5%).

Regarding adverse effects, 82.8% (679/820) of patients experienced adverse effects, and 19.6% (133/679) of these patients disrupted their treatment because of these effects without consulting with their doctors.

In terms of the information on the medications, 73% (599/820) of patients reported having received information from their doctors about treatment benefits, but only 44.6% (366/820) of patients had been informed about possible adverse effects for each medication. Patients with cardiovascular diseases tend to receive explanations on the benefit and health impacts of all their treatments more often than patients with mental disorders (317/403, 78.6% vs 195/292, 66.8%; *P*≤.001). Furthermore, 35.8% (294/820) of patients had only received partial information regarding the benefits for health and potential interactions and adverse effects associated with their medication. Regarding specific medications, 52.4% (154/294) of patients were informed about most of their medications, 47.6% (140/294) of patients received information about some medications, and 20% (160/820) of patients did not receive any information. Moreover, 26.2% (215/820) of patients were unaware of possible pharmaceutical interactions, and only 40.1% (329/820) of patients were aware of the risks of potential interaction between medications.

### Treatment Adherence

#### Perceived and Observed Treatment Adherence

In this study, the criteria for perfect medication adherence included taking all medication on time and as prescribed, without any interruptions. Overall, patients tended to perceive themselves to be more adherent than they actually were; 70.7% (580/820) of patients perceived themselves to be highly adherent (mean 8.9, median 9.9), 66.3% (544/820) reported taking their medication late or early, and 26.4% (217/820) regularly disregarded the prescribed dose. Patients with cardiovascular diseases perceived themselves to be highly adherent more often than those with mental disorders (320/403, 79.4% vs 173/292, 59.2%; *P*≤.001). Furthermore, many patients stopped or interrupted a medication either unintentionally (429/820, 52.3%) or intentionally (254/820, 31%) without consulting with a doctor. The most common reasons provided by the 646 patients who did not take their medication as prescribed were that they had forgotten to take it (335/646, 51.9%) or that they wanted to avoid experiencing adverse effects (188/646, 29.1%). Patients with mental disorders were more likely to intentionally disrupt their treatment than those with cardiovascular diseases (151/260, 58.1% vs 97/284, 34.1%; *P*≤.001), primarily because they wanted to avoid experiencing adverse effects (84/260, 32.3%). When patients with cardiovascular diseases intentionally disrupted their treatment, they also most often mentioned that they did so to avoid experiencing adverse effects (63/284, 22.2%).

#### Factors Associated With Treatment Adherence

Factors associated with treatment adherence included demographic characteristics, disease type, treatment characteristics, and patient awareness of treatment and the associated adverse effects (Table 3). Men were more adherent than women (152/278, 54.7% vs 204/542, 37.6% mostly perfectly adherent patients; *P*≤.001). The older patients (≥65 years) were markedly more adherent than the younger patients (112/189, 59.3% vs 244/631, 38.7% mostly perfectly adherent patients; *P*≤.001). The more complex the treatment regimen (eg, multiple treatments during the day, frequent dose changes), the less adherent the patients were to the treatment regimen (mostly perfectly adherent patients: 75/269, 27.9% simple treatment vs 62/347, 17.9% complex treatment; *P*≤.001). We also found that patients with cardiovascular diseases were more adherent than patients with mental disorders (228/403, 56.6% vs 81/292, 27.7% mostly perfectly adherent patients; *P*≤.001).

Patients who were less adherent were more likely to think that they would experience adverse effects when they started a new medication (126/216, 58.3% of poorly adherent patients vs 70/174, 40.2% of perfectly adherent patients; *P*≤.001). Similarly, the less adherent patients were to the medication, the more likely they were to worry about treatment constraints (160/216, 74.1% of poorly adherent patients vs 90/174, 51.7% of perfectly adherent patients; *P*≤.001). We also found that poorly adherent patients were less likely to receive information regarding treatment benefits and adverse effects for each medication (74/216, 34.2% of poorly adherent patients vs 102/174, 58.6% of perfectly adherent patients; *P*≤.001; Table 3).

**Table 3:** Factors associated with treatment adherence according to adherence level among patients with mental disorders or cardiovascular diseases or both conditions who were recruited for the French Carenity platform study (N=820).

Factors		Total	Poorly adherent, n (%)	Partially adherent, n (%)	Mostly adherent, n (%)	Perfectly adherent, n (%)	*P* value		
**Gender**								≤.001	
	Female	542	166 (30.6)	172 (31.7)	100 (18.5)	104 (19.2)			
	Male	278	50 (18)	76 (27.3)	82 (29.5)	70 (25.2)			
**Age group (years)**								≤.001	
	≥65	189	27 (14.3)	50 (26.5)	46 (24.3)	66 (34.9)			
	<65	631	189 (30)	198 (31.4)	136 (21.6)	108 (17.1)			
**Disease type**								≤.001	
	Cardiovascular diseases only	403	63 (15.6)	112 (27.8)	109 (27)	119 (29.5)			
	Mental disorders only	292	115 (39.4)	96 (32.9)	49 (16.8)	32 (11)			
**Complexity of treatment regimen**								≤.001^a^	
	Simple	269	58 (21.6)	75 (27.9)	61 (22.7)	75 (27.9)			
	Intermediate	204	52 (25.5)	61 (29.9)	54 (26.5)	37 (18.1)			
	Complex	347	106 (30.5)	112 (32.3)	67 (19.3)	62 (17.9)			
**Fear of treatment risks**								≤.001	
	No	125	17 (13.6)	23 (18.4)	39 (31.2)	46 (36.8)			
	Yes	695	199 (28.6)	225 (32.4)	143 (20.6)	128 (18.4)			
**Worry about treatment constraints**								≤.001	
	No	262	56 (21.4)	72 (27.5)	80 (30.5)	84 (32.1)			
	Yes	528	160 (30.3)	176 (33.3)	102 (19.3)	90 (17.1)			
**Received information on treatment benefits and impact on health**								≤.001	
	Yes	599	126 (21)	188 (31.4)	144 (24)	141 (23.6)			
	Yes partially	142	64 (45.1)	42 (29.6)	21 (14.8)	15 (10.5)			
	No	79	26 (32.9	18 (22.8)	17 (21.5)	18 (22.8)			
**Received information on adverse effects**								≤.01	
	Yes	660	162 (24.5)	208 (31.5)	148 (22.4)	142 (21.5)			
	No	160	54 (33.7)	40 (25)	34 (21.3)	32 (20)			

^a^The *P* value only refers to the comparison of the simple and complex treatment regimens.

#### Reporting of Adherence Issues

Among the patients who did not always take their medication as prescribed, 59.1% (382/646) did not consult with their physician because they did not think it was necessary (146/382, 38.2%), they forgot (116/382, 30.4%), or they were afraid of disappointing their doctors or of being judged (87/382, 22.8%). Patients with the most risk factors (ie, overweight, smoking, age ≥65 years) seemed least likely to always notify their physician of adherence issues (26/81, 32.1% of high-risk patients vs 162/363, 44.6% of low-risk patients; *P*=.05).

#### Solutions and Tools Used by Patients

Just over half of respondents (428/820, 52.2%) used at least one tool to help them properly adhere to their treatment, such as a pill organizer (334/820, 40.7%) or an alarm (80/820, 9.7%). Only 1.9% (16/820) of patients used a smartphone application. Patients with cardiovascular diseases were more inclined to use a tool than those with mental disorders (224/403, 55.6% vs 136/292, 46.6%; *P*=.02). A total of 12.2% (100/820) of patients reported that they received help with their medication from a caregiver. Among those who received assistance, the caregiver primarily reminded them to take their medication (60/100, 60%) or purchased the medication for them (43/100, 43%).

### Patient Behavior Profiles

#### Patients With Cardiovascular Diseases Only

Patients with cardiovascular diseases were classified as follows based on 3 identified behavior profiles: at-risk patients, tolerant patients, and reporter patients.

The at-risk patients (168/403, 41.7%) were the least adherent (110/168, 65.5% were not fully adherent). The majority of these patients were women (121/168, 72%). Although these patients perceived themselves to be adherent (123/168, 73.2%), they took ≤3 pills per day (71/168, 42.3%), used complementary approaches (138/168, 82.1%), and intentionally deviated from initial prescriptions (65/140, 46.4% of these patients did not take their medication as prescribed). These patients were worried about the treatment interfering with their daily routine (55/168, 32.7%) and the frequency of administration (68/168, 40.5%). Moreover, 33.9% (57/168) of the at-risk patients were more likely to worry about becoming dependent on their medication, and 49.4% (83/168) of patients worried about medication efficacy. These patients visited their physician more often (139/168, 82.7%) but did not always notify them of nonadherence (88/168, 52.4%) because of a fear of disappointing them (80/88, 47.86%). Finally, 73.2% (123/168) of these patients did not receive information about medication benefits, and 75% (126/168) of patients had discussed constraints and risks with someone.

The reporter patients (145/403, 36%) were largely adherent (116/145, 80%) and perceived themselves to be adherent (131/145, 90.3% of these patients perceived themselves to be fully adherent). The majority of these patients primarily included older patients (66/145, 45.5% ≥65 years of age) and men (90/145, 62.1%) who took >3 pills per day (118/145, 81.4%). They also believed that they were well-informed (122/145, 84.1%), they used practical tools to manage their medications (91/145, 62.7%), and they were aware of potential adverse interactions between their medication and tobacco (54/145, 37.2%) and alcohol (74/145, 51%).

The tolerant patients (90/403, 22.3%) experienced fewer adverse effects (78/90, 86.7%) and did not adjust their treatment (85/90, 94.4%). These patients were less aware of adverse interactions between their treatment and other medications (66/90, 73.3%) and between their treatment and alcohol (58/90, 64.4%). These patients did not inform their doctors about adherence issues (32/42, 76.2%).

#### Patients With Mental Disorders Only

Patients with mental disorders were grouped as follows based on 3 identified behavior profiles: fearful patients, at-risk patients, and confident patients.

The fearful patients (125/292, 42.8%) were polymedicated (85/125, 68% of patients took ≥3 pills per day), had experienced treatment adverse effects (125/125, 100%), and anticipated adverse effects when they started a treatment (75/125, 60%). These patients were concerned about treatment risks (101/125, 80.8%) and adjusted their treatment in the event of adverse effects (55/125, 44%); 25.6% (32/125) stopped the medication and 8.8% (11/125) modified the dose. These patients also used fewer complementary approaches such as nonpharmaceutical alternatives (54/125, 43.2%), homeopathy, (16/125, 12.8%), and acupuncture (3/125, 2.4%).

The at-risk patients (102/292, 34.9%) were the least adherent patients (82/102, 80% of these patients were not fully adherent), and the majority of these patients were women (90/102, 88.2%). They took only 1-2 pills per day (57/102, 55.9%) and perceived themselves to be poorly adherent (55/102, 53.9%). These patients worried about treatment risks and constraints (82/102, 80.4%). All patients used at least one other product or an alternative (102/102, 100%); nearly all patients (94/102, 92.1%) used nonpharmaceutical therapy, 89.2% (91/102) of patients used homeopathy, and 52% (53/102) used acupuncture. They experienced adverse effects (102/102, 100%) and adjusted their treatment in case of an adverse effect (45/102, 44.1%); 17.6% (18/102) stopped the medication and 14.7% (15/102) modified the dose. Of these patients, 75.5% (77/102) reported adverse effects to their physician and 67.6% (69/102) had discussed constraints and risks with their pharmacist.

The confident patients (65/292, 22.3%) were more adherent (27/65, 42%) and the majority of those who were adherent were men (44/65, 67.7%). Few patients experienced adverse effects (44/65, 67.7% did not experience adverse effects); these patients did not adjust their treatment in the event of an adverse effect (60/65, 92.3%) and they were less likely to use complementary approaches (27/65, 41.4%). These patients worried less about the risks associated with their medication (22/65, 33.8%). They were less informed about risks (21/65, 32.3%) but well informed about treatment benefits (50/65, 76.9%). Finally, among the confident patients who did not always notify their doctors in the event of therapeutic nonadherence, 42.8% (15/35) did not think it was necessary to do so.

## Discussion

### Principal Findings

Web-based communities represent an increasingly popular and accessible platform for patients to learn about their condition and participate in clinical studies [22]. These web-based patient communities also provide researchers access to data about specific patient populations that are demographically representative. These tools enable the assessment of data concerning patient behavior, experiences, and well-being in all aspects of their lives (medical, professional, and personal), which are difficult to observe using other methods [22].

The current study focused on perceptions of iatrogenic risk and treatment adherence in patients with cardiovascular diseases, mental disorders, or both cardiovascular diseases and mental disorders. All patients were registered on the largest web-based Carenity patient community in France. We found that 82.8% (679/820) of patients experienced adverse effects associated with their medication. While the majority of patients (492/679, 72.4%) informed their doctor about adverse effects, many patients took steps to address adverse events on their own, with 30.8% (209/679) disrupting their treatment without medical advice. These results indicate that adverse effects present an understandable challenge for patients [23] and represent a major barrier to medication adherence [24]. We also found that well-informed patients were more likely to report adverse effects to a health care professional and are less likely to disrupt their treatment on their own. The frequent report of adverse events likely explains why most patients anticipated adverse effects when they started a new medication and worried about treatment risks and constraints such as dependence, lack of efficacy, effects on another condition, and intolerance.

Regarding therapeutic adherence, patients tended to perceive themselves to be more adherent than they actually were. Medication adherence is often overestimated by patients [25]. In this study, more than half the number of patients (426/820, 52%) unintentionally stopped or interrupted treatment at least once, often because they forgot or wanted to avoid experiencing adverse effects.

The results are consistent with those of previous studies that have indicated that approximately 50% of patients undergoing long-term therapy were nonadherent to their treatment [9,11]. A separate study has also indicated that forgetting to take medication and having limited awareness were the most frequently cited reasons for treatment nonadherence [14,26]. Previous studies have also shown that several factors are associated with adherence, including patient characteristics (gender, age, ethnicity, marital status), symptom intensity, medication type, route of administration, and severity of adverse effects [27,28]. In this study, the factors associated with treatment adherence included gender, age, treatment complexity, and disease type. Men were more adherent than women, and patients ≥65 years of age were significantly more adherent than the younger patients. Moreover, patients with a simple treatment regimen and those who had received information about treatment benefits and potential adverse effects tended to be more adherent. The degree of adherence also varied between the 2 disease groups, as patients with cardiovascular diseases tended to be more adherent than those with mental disorders. Less adherent patients were less aware of potential drug interactions, although they were more likely to anticipate adverse effects when they started a new treatment. They also worried more about risks of treatment dependency, lack of treatment efficacy, and the constraints that therapy may impose on their daily routine.

It is important to emphasize that the study was performed during the ongoing COVID-19 pandemic. To date, limited research is available on how the pandemic has specifically affected therapeutic adherence in NCD patients, but it is well known that the COVID-19 pandemic has disrupted health care services around the world, which has possible implications on therapeutic adherence in patients. One systematic review reported a significant failure of patients with inflammatory bowel disease to adhere to therapies during the COVID-19 pandemic [29]. However, as shown in a pharmacoepidemiological study conducted using data from the French National Health Data System, the COVID-19 pandemic did not seem to lead to a shortage of treatment for patients with cardiovascular diseases. For example, a significant number of hypertensive patients overstocked their medication when the lockdown was announced. On the other hand, the study demonstrated a decrease in consumption of nonprescription drugs and products needed for examinations, such as colonoscopies, or contrast agents. Therefore, the combination of factors related to social restriction (lockdowns, difficulty in accessing health care or treatments) and patient-related factors (fear of infection, decision to take or not take the drug, treatment dosage adjustment) may have impacted therapeutic adherence in NCD patients, even though this impact seems to be more nuanced in France than in other countries [30].

The 3 patient profiles identified for each disease type provide significant insight for improving therapeutic adherence and adjustment strategies specifically according to patient behaviors. With respect to the cardiovascular disease profiles, the at-risk patients, who were less adherent and worried about treatment risks and adverse effects, should be better informed about treatment risks (especially adverse effects) and treatment benefits. The tolerant patients, who were polymedicated, more adherent, and less worried about risks and adverse effects, should be better informed about tools they can use to properly manage their medication. The reporter patients, who did not discuss adverse effects with their pharmacists and were unaware of treatment interactions, should be better informed about the risks of treatment interactions and encouraged to notify their doctors when medications are not taken as prescribed.

Regarding the mental disorder profiles, the at-risk patients, who were not adherent, rarely communicated with their doctors, and overestimated treatment risks and constraints, should have access to specialized therapeutic educational programs to improve awareness and medication adherence (eg, shared decision making) [31]. The fearful patients, who were polymedicated, experienced adverse effects, and were better informed about drug interactions and adverse effects, should be closely monitored by their doctors so that advice focused on their specific needs can be provided. Finally, the confident patients, who tended not to seek assistance when needed and did not present major challenges, should be encouraged to build a better relationship with their doctors and seek assistance when necessary.

### Limitations

A few study limitations should be mentioned. As the study was based on data collected via a web-based survey, it may exclude patients who are not comfortable using or do not have access to internet or a computer or who are non-French speakers living in France. The underrepresentation of older patients in the Carenity community and the overrepresentation of patients who are actively concerned about their health may have also led to selection bias. Moreover, patient characteristics and medication adherence were assessed using self-reported measures, which may have led to recall bias. Finally, the group of patients with both cardiovascular diseases and mental disorders could not be included in the statistical analysis because of the small number of patients in the group. Patients with multiple NCDs should be included in future studies because they are at an increased risk of iatrogenic disease [18].

Patients completed the questionnaire without the guidance of their physicians, so desirability bias was greatly limited. As individuals registered in patient communities may likely be heavily burdened by their condition, our study population may overrepresent highly symptomatic and polymedicated patients. Nevertheless, it has been shown that characteristics of patients in Carenity communities reflect the main characteristics of web-based users willing to share their medical experience but with an overrepresentation of female patients aged 25-54 years [32]. This study exclusively included patients registered on the French Carenity platform and did not include other relevant sampling procedures and methods. Despite the fact that study recruitment focused on a homogeneous population of patients through the Carenity platform, the results of the study are not generalizable to the larger population of patients with these disorders.

### Conclusions

Overall, the results of this web-based survey study provide important insight into patients’ perspectives and behaviors because the anonymous nature of the survey allowed patients to respond openly and honestly. These findings emphasize the importance of involving patients in medical decisions and providing patients with information about treatment benefits, treatment adherence, potential adverse effects and risks of treatment, and potential drug interactions. Therapeutic alliance significantly helps patients to understand both the disease aspect and the therapeutic options, thereby improving medication adherence and overall disease management [11,18]. Integrative and comprehensive patient care that considers the complementary therapeutic approaches used by patients could also improve medication adherence [26]. Our results also show that caregivers and pharmacists should be empowered to proactively support and better educate patients with an NCD who require multiple medications. Practical tools should be developed to remind patients to take their medication as prescribed, and additional studies should assess improved support strategies for patients with chronic diseases.

Classical adherence studies are often biased and do not accurately represent the patient population for a given disease. Web-based platforms on which patients can share their medical experiences in an anonymous manner may provide unique insight into the perspectives of patients undergoing therapy for an NCD, which could help to improve disease management and eventually prevent premature deaths.

## Abbreviations

NCD: noncommunicable disease
